# Prenatal Alcohol Exposure Modifies Glucocorticoid Receptor Subcellular Distribution in the Medial Prefrontal Cortex and Impairs Frontal Cortex-Dependent Learning

**DOI:** 10.1371/journal.pone.0096200

**Published:** 2014-04-22

**Authors:** Andrea M. Allan, Samantha L. Goggin, Kevin K. Caldwell

**Affiliations:** Department of Neurosciences, School of Medicine, University of New Mexico Health Sciences Center, Albuquerque, New Mexico, United States of America; Florida International University, United States of America

## Abstract

Prenatal alcohol exposure (PAE) has been shown to impair learning, memory and executive functioning in children. Perseveration, or the failure to respond adaptively to changing contingencies, is a hallmark on neurobehavioral assessment tasks for human fetal alcohol spectrum disorder (FASD). Adaptive responding is predominantly a product of the medial prefrontal cortex (mPFC) and is regulated by corticosteroids. In our mouse model of PAE we recently reported deficits in hippocampal formation-dependent learning and memory and a dysregulation of hippocampal formation glucocorticoid receptor (GR) subcellular distribution. Here, we examined the effect of PAE on frontal cortical-dependent behavior, as well as mPFC GR subcellular distribution and the levels of regulators of intracellular GR transport. PAE mice displayed significantly reduced response flexibility in a Y-maze reversal learning task. While the levels of total nuclear GR were reduced in PAE mPFC, levels of GR phosphorylated at serines 203, 211 and 226 were not significantly changed. Cytosolic, but not nuclear, MR levels were elevated in the PAE mPFC. The levels of critical GR trafficking proteins, FKBP51, Hsp90, cyclophilin 40, dynamitin and dynein intermediate chain, were altered in PAE mice, in favor of the exclusion of GR from the nucleus, indicating dysregulation of GR trafficking. Our findings suggest that there may be a link between a deficit in GR nuclear localization and frontal cortical learning deficits in prenatal alcohol-exposed mice.

## Introduction

Optimal levels of glucocorticoid-dependent signaling are necessary for learning and attention. Dysregulation within glucocorticoid signaling pathways alters habitual responding [1] and can affect decision making. In a series of studies, Gourley and colleagues [2] demonstrated that glucocorticoid receptor (GR) signaling in the medial prefrontal cortex (mPFC) is necessary for adaptive responding and flexible decision making strategies, particularly under stressful conditions. Glucocorticoids, acting via the GR, exert a wide array of actions both in the periphery and in the brain, including regulation of neural pathways connecting the mPFC with the hippocampus and the amygdala [3]. Activation of the GR within the mPFC alters the set point of the hypothalamic–pituitary–adrenal (HPA) axis [3].

Many, but not all [4], of the effects of the glucocorticoids are mediated by GR-dependent control of gene transcription [5]. In the ligand-unbound state, the GR is localized to the cell cytosol; upon ligand binding, the GR translocates to the nucleus [6]. Trafficking of the GR between these cellular compartments is controlled by multiple proteins, including FK506-binding proteins 51 (FKBP51; FKBP5) and 52 (FKBP52, FKBP4), heat shock protein 90 (Hsp90), cyclophilin 40, dynein and dynamitin [7].

Exposure to alcohol *in utero* produces a range of morphological and neurocognitive outcomes in offspring collectively called fetal alcohol spectrum disorders (FASDs) [8]. Children with FASD exhibit an array of neurocognitive deficits including diminished learning and memory, poorer motor function and executive functioning deficits [9]. Deficits in executive functioning that are displayed by FASD children include a heightened focus on one particular task, or a small component of a task, leading to a failure to assimilate other important contingent information. In addition, an inability to alter responding to a change in stimulus-response associations (sometimes called perseveration) is a hallmark on neurobehavioral assessment tasks for human FASD [9]. While it is well known that the neural pathways underlying these behaviors critically involve the frontal cortex, the molecular substrates underlying FASD-associated deficits in cognitive flexibility and response inhibition are poorly understood.

Studies employing rodent models of FASD have primarily utilized behavioral tasks that rely on associative learning and, thus, have focused on assessments of hippocampal functioning. A few rodent behavioral tasks are known to assess frontal cortex-dependent cognitive performance: such as, cognitive flexibility, discrimination reversal learning, extinction learning and set shifting. Prenatal alcohol exposure (PAE) has been shown to attenuate extinction of previously reinforced behavior [10] and produce equivocal results on reversal learning [11]. While both human and rodent studies have indicated that GR signaling is necessary for new response outcome learning [1], to date, no PAE rodent model has assessed glucocorticoid signaling within the mPFC. We focused our studies on adolescence, as it is a dynamic period of frontal cortical growth and development [12], which may program future adult behavioral contingencies. In male rodents adolescence extends from approximately postnatal day (PD) 28 to PD 60 [13]. In our recent work [14], we reported that PAE was associated with increased GR nuclear localization in the adolescent mouse hippocampal formation, indicating that the trafficking of cytosolic GRs to the nucleus may be increased as the result of PAE. In the studies presented here we explore the impact of PAE on behavior that is dependent on mPFC performance and on GR subcellular distribution in the mPFC.

## Materials and Methods

### Animals

#### Ethics statement

All procedures described were approved by the University of New Mexico Institutional Animal Care and Use Committee (IACUC). C57BL/6J mice (The Jackson Laboratory, Bar Harbor, ME) were maintained in a reverse light/dark (lights off at 0800 hr and on at 1800 hr), temperature-controlled vivarium and housed with same sex littermates. All behavioral studies were conducted between 0900 and 1300 hr under dim red illumination. At the time of tissue collection or at the beginning of behavioral testing, all animals were PD 40–50. All tissue was taken between 0700 and 0800 hr. Naïve male mice were used in the all of the studies presented.

### Prenatal alcohol exposure

The PAE paradigm has been described previously [15]. Alcohol consumption (9.5±1.3 g ethanol/kg body weight/4 hr) was similar to that reported in our recent studies [15], which yielded average blood alcohol concentrations of approximately 90 mg/dL after four hours of consumption. Briefly, C57BL/6J female mice had limited access to either 10% (w/v) ethanol in 0.066% (w/v) saccharin or saccharin 0.066% (w/v) alone (SAC) for 4 hr/d. Drinking was established prior to mating, maintained throughout gestation, and withdrawn following parturition using a step-down procedure over a six-day period. Offspring were weaned at 23 (±1) days of age.

### Reversal Learning

A total of 6 naïve male PAE and 6 naïve male SAC mice each from different litters were used in these studies. Mice were placed on food restriction and maintained at 85% starting weight. A Plexiglas Y-maze (runway 78 cm ×12 cm; y-arms 33 cm ×12 cm) was used for these studies. Mice were acclimated to the maze for 15 minutes on the day prior to training. Extraneous sounds were attenuated by a white noise generator. Mice were brought into the behavior testing room 30 min prior to placement in the maze. Maze runway and arms were cleaned between trials with 70% isopropyl alcohol.

Acquisition Training consisted of 5 trials per day for 6 days. One arm of the Y-maze was baited with Cheerios. Mice were placed at the end of the runway and permitted to enter the arms of the maze and locate the food reward. An initial selection of the baited arm was recorded as a correct choice and a percentage of correct choice was calculated for each training day. If the first arm choice was not correct (not baited), the mouse was permitted to exit the arm and enter the baited arm. Once they had consumed the food, they were returned to their home cage. The side placement of the food reward and the orientation of the maze within the room remained constant throughout the training period.

Reversal Testing followed immediately after acquisition training, beginning on day 7. Testing consisted of 5 trials per day for 6 days. The food reward was located in the alternate arm (arm not baited during the acquisition training). The mouse was placed at the end of the runway and permitted to choose to enter an arm of the maze. Once an arm was entered, the non-selected arm was manually blocked off using a Plexiglas guillotine door. A visit to the baited arm was recorded as a correct choice and a percentage of correct choice was calculated for each reversal testing day.

### Isolation and subcellular fractionation of medial frontal cortex tissue

Medial prefrontal cortex (comprised of the pooled cingulate cortex, infralimbic, and prelimbic areas) was mechanically dissected and immediately frozen in liquid nitrogen until use in the studies. The dissection involved a coronal slice made between bregma 2.1 mm and 1.34 mm (interaural 5.90 mm to 5.14 mm) and a wedge of tissue from the central hemispheric fissure down to the midline of the corpus callosum was taken on ice. Subcellular fractions (nuclear and cytosolic) were prepared as described in Buckley and Caldwell [16] except buffers also contained 20 mM β-glycerophosphate, 20 mM sodium pyrophosphate, and 10 mM sodium fluoride. Dynein intermediate chain 1 and 2 were evaluated using a post-nuclear lysate prepared as described in [14] and Dynamitin was evaluated in a Trition –X100 -soluble membrane fraction prepared as described in Buckley and Caldwell [16] but modified using 1 mM Triton X100.

### Immunoblotting

Gel electrophoresis and immunoblotting were conducted using established protocols [14]. PAE and SAC samples were run on the same blots for each target protein examined. Total GR and phosphorylated isoforms of GR were assessed in mPFC nuclear (8 µg) and cytosolic (20 µg) subcellular fractions. Anti-MR immunoreactivity was measured in cytosolic (10 µg) and nuclear (15 µg) preparations. 8 µg cytosolic fraction was used for the anti-FKBP51 blot and 1 µg cytosolic fraction was loaded for the anti-FKBP52 blot. Dynein intermediate chain 1 and 2 were evaluated using 12 µg lysate. Dynamitin was analyzed in 10 µg membrane fraction. Samples were electrophoresed then transferred to PVDF membrane (Bio-Rad Laboratories, Hercules, CA; #162–0177 or Immobilon-FL, EMD Millipore Corporation, Billerica, MA). Membranes were stained with Coomassie Brilliant Blue R-250 (Bio-Rad; #161–0400) to assess and correct for the amount of protein loaded per lane. For the anti-GR, anti-phospho-GR, anti-FKBP51 and anti-FKBP52 blots, Coomassie staining was performed on regions of the blot that were immediately above and/or below the region of interest; for all other blots, the entire blot was stained and then destained. Membranes were then blocked for 1 hr in 0.25% (w/v) Tropix I-BLOCK (Applied Biosystems, Grand Island, NY; #T2015) or Odyssey Blocking Buffer (LI-COR Biosciences, Lincoln, NE; #927–40000) and incubated overnight in primary antibody as described below.

For the Hsp90, Cyclophilin 40, Dynamitin and Dynein IC1/2 studies the following primary antibodies and dilutions were used: Hsp90 (1∶500, Cell Signaling #4875S; Danvers, MA), cyclophillin 40 (1∶500, Abcam #ab3562; Cambridge, MA), Dynamitin (1∶500, Abcam #ab56687), and Dynein IC1/2 (1∶500, Santa Cruz #sc-66866). Dynein IC1 and IC2 bands were distinguished by molecular weight (IC1: 74 kDa and IC2: 72 kDa). Goat anti-rabbit and goat anti-mouse secondary antibodies were used at 1∶30,000 dilution, as described in [14]. Immunoreactivities were quantified using Quantity-One 1-D Analysis Software (Bio-Rad Laboratories). The linear protein concentration range was determined for each antibody.

Quantitative Western blotting for assessment of GR, phosphorylated forms of GR, FKPB51, FKBP52 and mineralocorticoid receptor (MR) was performed using two-channel infrared direct detection (Odyssey Imaging System, LI-COR Biosciences). Primary antibodies and dilutions were as follows: anti-GR (1∶100, Santa Cruz GR (A-4) #sc-376425), anti-phospho-serine 226 GR (1∶1,000, Assay Biotechnology #A0432; Sunnyvale, CA), anti-phospho-serine 211 GR (1∶1,000, Cell Signaling Technology #4161), anti-phospho-serine 203 GR (1∶1,000, biorbyt # orb105971; San Francisco, CA), anti-FKBP51 (1∶1,000, Abcam #2901) anti-FKBP 52 (1∶200, Santa Cruz #sc-1803) and anti-MR (1∶500, Santa Cruz #sc-11412). All primary antibodies were diluted in PBS-T (1.06 mM KH_2_PO_4_, 2.97 mM Na_2_HPO_4_, 155 mM NaCl, 0.1% (v/v) Tween-20, pH 7.4). For the GR/phospho-GR studies, the secondary antibodies (LI-COR Biosciences) were IRDye 680RD goat (polyclonal) anti-mouse IgG (catalog #926–68070) and IRDye 800CW goat (polyclonal) anti-rabbit IgG (catalog #926–32211); both antibodies were diluted to 0.1 µg/mL in PBS-T. IRDye 800CW goat (polyclonal) anti-rabbit IgG secondary antibody diluted to 0.1 µg/mL in PBS-T was also used in the MR studies. For the FKBP51 and FKBP52 studies, the secondary antibodies (LI-COR Biosciences) were donkey anti-rabbit IgG (catalog #926–68073, IRDye 680RD) and donkey anti-goat (catalog m#926–32214, IRDye 800CW) both used at 0.067 µg/mL in PBS-T. Blots were quantified using Image Studio (version 3.1 LI-COR Biosciences).

All blots were corrected to their respective Coomassie stain. Coomassie staining was demonstrated to be linear (Figure S1 in File S1) within the range of protein concentrations used in these studies and was not altered by the prenatal treatment in any of the subcellular and tissue fractions tested (Figure. S2 A–D in File S1). For each of the quantitative immunoblots determinations, a total of 7–10 PAE and 7–10 SAC naïve mice each from a different litter were tested. Three representative immunoblots for each prenatal condition are presented in the figures.

### Data analysis

Mixed model repeated measures ANOVA was conducted for the Y-maze reversal learning task. To compare the two prenatal conditions for the immunoblotting studies, independent Student's t-tests were employed (GraphPad Software, v.4.03; San Diego, CA). The level of each phospho-GR isoform was expressed relative to the level of the total GR present in the sample using the following method. Three immunoblots were prepared from the same aliquot of a sample; each was probed for one of the three phospho-GR isoforms and for total GR. The anti-phospho-GR and anti-total GR immunoreactivities were determined and these values were used to calculate the ratio of the anti-phospho-GR immunoreactivity/anti-total GR immunoreactivity.

## Results

### PAE mice display an inflexible response in reversal learning task

**Figure 1:**
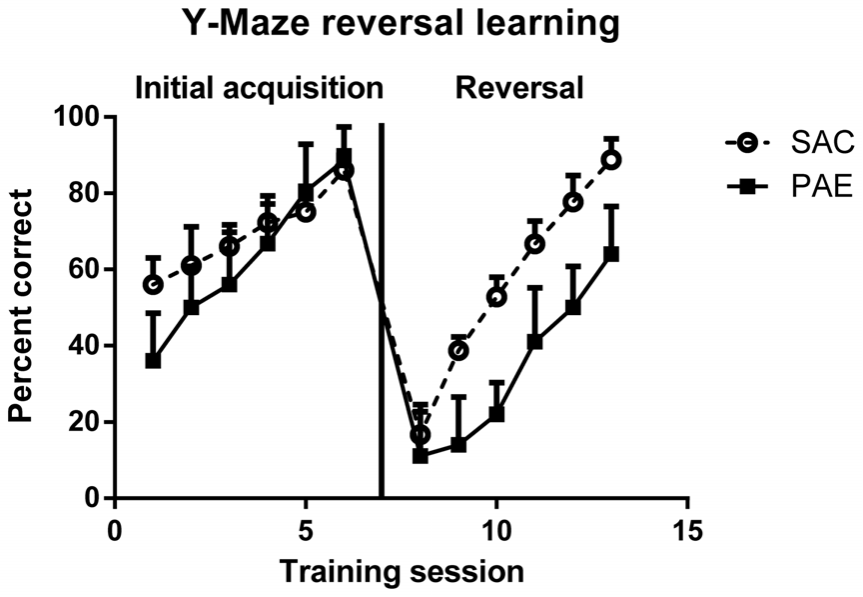
Y- Maze Reversal learning performance in naïve male 45–50 day old prenatal alcohol exposure (PAE, n = 6) and saccharin control (SAC, n = 6) mice. Data presented are mean percent correct arm choice (±SEM). Details of the methods for these procedures are presented in Materials and Methods. Analyses are presented in the Results section.

The Y-maze reversal learning task was analyzed separately for the two phases, initial acquisition phase and the reversal phase (Figure 1). Performance of both groups of mice improved with repeated trial sessions [F (1,20) = 37.5, p<.0001] and there was an overall difference between the PAE and SAC in Y-maze reversal learning [F(1,20) = 18.8, p<.0001]. An interaction between prenatal treatment and training condition (acquisition or reversal) was significant [F (1,20) = 5.95, p = .024], as the PAE were slower to learn during the reversal phase of the task.

### PAE mice have reduced GR nuclear localization but phospho-GR levels were not altered

We have reported that PAE increased nuclear localization of the GR in the adolescent male mouse hippocampal formation [14]. We sought to determine if there was an effect of PAE on GR nuclear localization in the mPFC. Additionally, we assessed the phosphorylation status of the GR, as phosphorylation of the receptor impacts both GR stability [17] and GR interaction with transcription factor complexes [18]. We determined the effects of PAE on the level of GR phosphorylation at three sites that are within the N-terminal activation function 1 (AF1) domain of the receptor, which is critical for transcriptional regulation and transactivation [18], [19].

**Figure 2:**
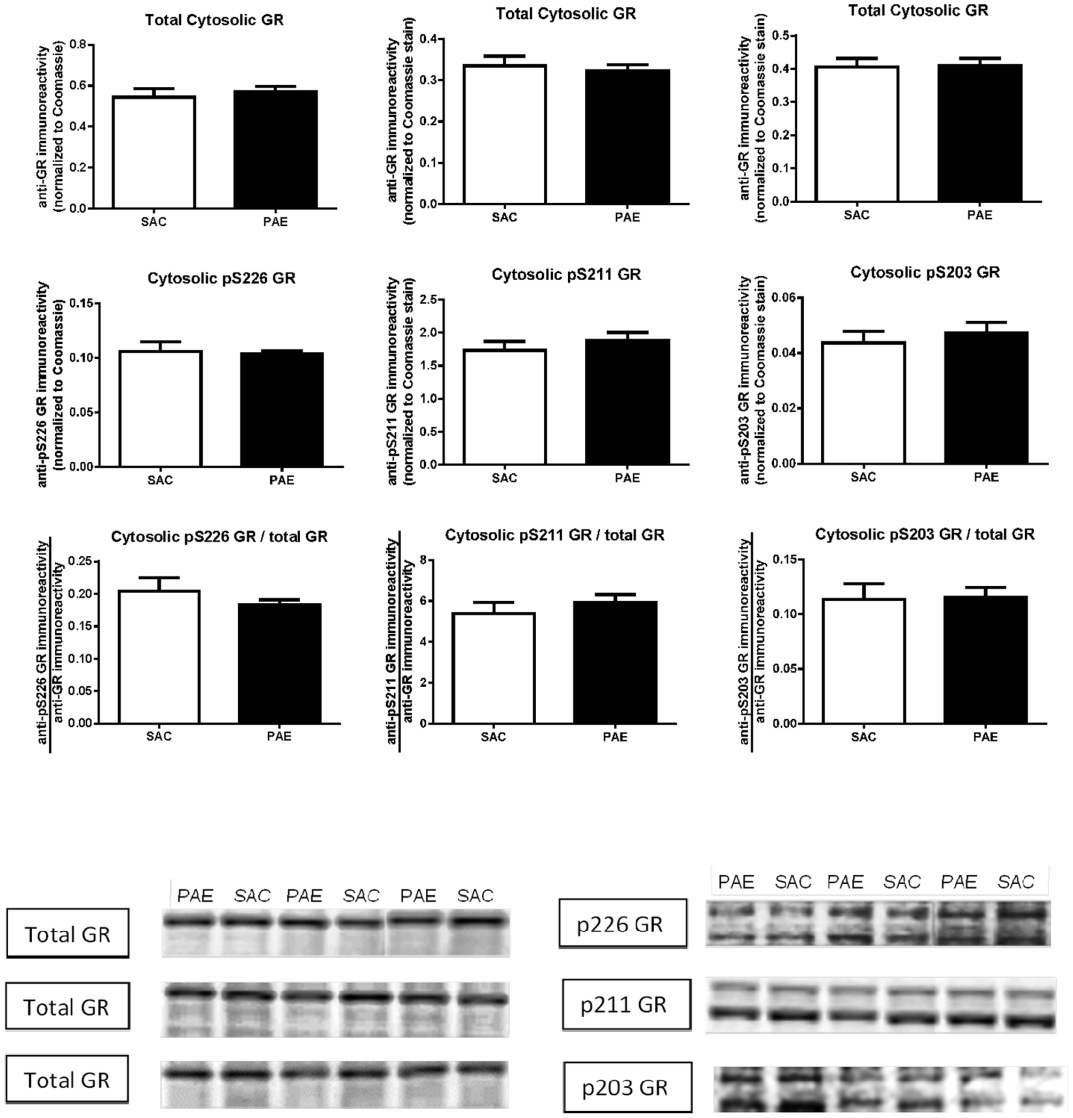
Cytosolic levels of the glucocorticoid receptor (GR), phospho-serine 226 (pS226) GR (left column), phospho-serine 211 (pS211) GR (center column) and phospho-serine 203 (pS203) GR (right column) in the medial frontal cortex of saccharin (SAC) control and prenatal alcohol exposure (PAE) offspring. Cytosolic fractions were prepared and anti-GR (top row graphs) and anti-phospho-specific GR (middle row graphs) immunoreactivities were determined as described in the Materials and Methods. Anti-total GR and anti-phospho-GR data are presented as the mean (± SEM) immunoreactivities corrected to Coomassie stain. The ratio of the anti-phospho-GR to anti-total GR immunoreactivities (bottom row graphs) was calculated as described in the Materials and Methods. The levels of total GR and all three phospho-GRs, as well as the ratios of all three phospho-GR/total GR, were not different in SAC (n = 7) and PAE (n = 7) mice. Representative Western blots are presented below.

**Figure 3:**
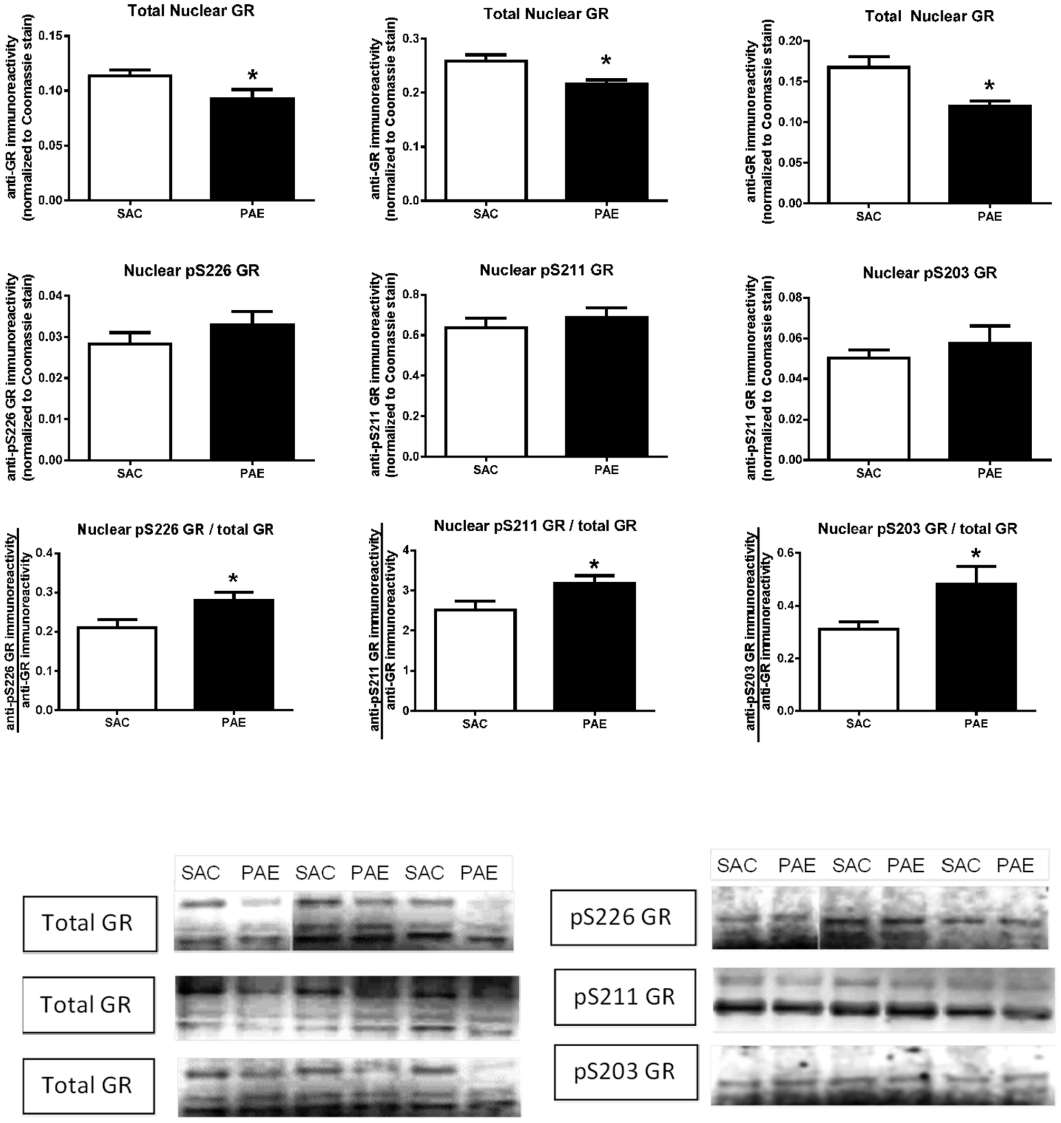
Nuclear levels of the glucocorticoid receptor (GR), phospho-serine 226 (pS226) GR (left column), phospho-serine 211 (pS211) GR (center column) and phospho-serine 203 (pS203) GR (right column) in the medial frontal cortex of saccharin (SAC) control and prenatal alcohol exposure (PAE) offspring. Nuclear fractions were prepared and anti-GR (top row graphs) and anti-phospho-specific GR (middle row graphs) immunoreactivities were determined, as described in the Materials and Methods. Anti-GR and anti-phospho-GR data are presented as the mean (± SEM) immunoreactivities from PAE (n = 9–10) and SAC (n = 9–10) mice corrected to Coomassie stain. The ratio of the anti-phospho-GR to anti-total GR immunoreactivities (bottom row graphs) was calculated as described in the Materials and Methods. Total nuclear GR levels were significantly lower in the PAE mice (p = .05 for pS226 GR, left top panel; p = .008 for pS211 GR, center top panel; p = .005 for pS203 GR, right top panel). The ratio of each phospho-GR to total GR was significantly higher in the PAE (pS226 GR/total GR, p = .04; pS211 GR/total GR, p = .04; pS203 GR/total GR, p = .02). Representative Western blots are presented below.

We measured anti-GR and anti-phospho-GR immunoreactivities in cytosolic (Figure 2) and nuclear (Figure 3) subcellular fractions isolated from SAC and PAE mice. Three anti-GR immunoreactive bands were detected in the nucleus (Figure 3, top row of representative immunoblots), while, in the cytosol (Figure 2, top row of representative immunoblots), a predominant anti-GR band, co-migrating with the slowest migrating band in the nucleus, was detected. We quantified all three bands of anti-total GR immunoreactivity in the nuclear fraction and used the sum of the bands for the value for each sample, while in the cytosolic fraction we quantified the primary, and essentially only, band that was detected. While cytosolic GR levels did not differ between SAC and PAE mice (top row Figure 2), a decrease in nuclear levels of GR (Figure 3 top right: t(17) = 3.16, p<0.01, Figure 3 top center; t(18) = 2.98, p<0.01, Figure 3 top left; t(15) = 2.15, p<0.05) was observed.

Seven phosphorylation sites have been identified in the mouse GR; five of these sites are conserved in the human GR [20]. Commonly, the phosphorylation sites are referred to using the human sequence amino acid numbering. We assessed the levels of phospho-serine 203 (pS203), phospho-serine 211 (pS211) and phospho-serine 226 (pS226) GR in the cytosolic and nuclear compartments.

The immunoblot images in Figure 2 show that, in the cytosolic fraction, the anti-pS203, anti-pS211 and anti-pS226 GR antibodies each detected two bands. In Figure 2, as well as Figure 3, the images for the anti-total GR and anti-phospho GR blots are of exactly the same dimensions so that the relative migrations of the anti-total GR and anti-phospho GR bands can be seen. For each of the anti-phospho GR antibodies, the upper (slower migrating) of the two anti-phospho GR immunoreactive bands co-migrated with the primary band recognized by the anti-total GR antibody. We quantified both anti-phospho GR bands and used the sum of the two bands for the value for each sample.

In the case of the nuclear fraction (Figure 3), we, again, detected two immunoreactive bands with the anti-pS211 GR antibody and quantified both bands. The blots of the anti-pS203 and anti-pS226 GR immunoreactivies both showed a primary band that co-migrated with the middle band detected with the anti-total GR antibody. We quantified this band and used the data for the anti-pS203 and anti-pS226 GR data shown in Figure 3. In the anti-pS203 and anti-pS226 GR immunoblots, we also detected a second band that migrated slightly faster than the fastest migrating of the three immunoreactive bands detected with the anti-total GR antibody. Although we think that it is likely that this band represents a GR isoform that does, indeed, contain pS203 or pS226, we are did not include it in our analyses.

Cytosolic (Figure 2, middle row) and nuclear (Figure 3, middle row) levels of each of these three phospho-GRs did not differ between SAC and PAE mice, demonstrating that GR phosphorylation states in PAE mPFC have been maintained at levels similar to SAC despite the reduction in total GR. The ratio of nuclear phospho GR to total GR proved to be significantly higher in the PAE relative to SAC (Figure 3 bottom row: pS226 GR (t(15) = 2.4, p = .03); pS211 GR (t(18) = 2.26, p = .04; pS203 GR (t(17) = 2.5, p = .024).

### PAE did not affect nuclear MR levels, although cytosolic levels of the MR were increased

**Figure 4:**
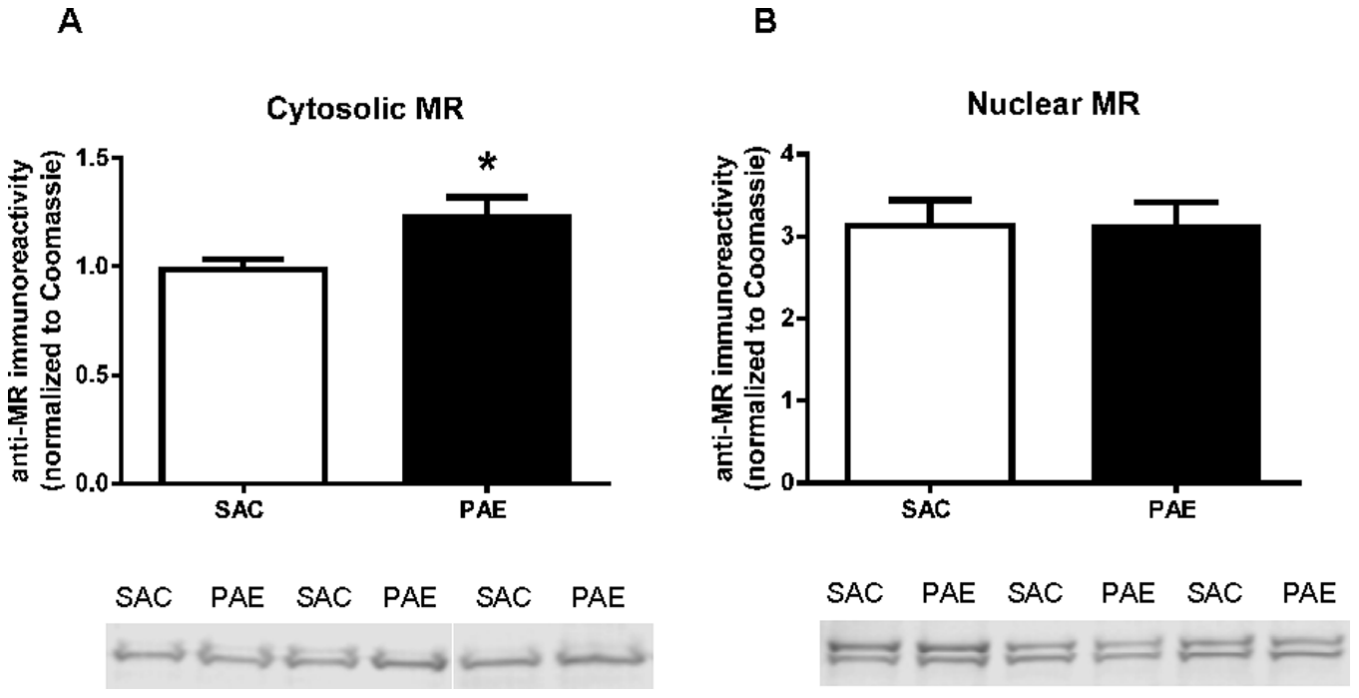
Cytosolic (A) and nuclear (B) levels of the mineralocorticoid receptor (MR) in the medial frontal cortex of saccharin (SAC) control and prenatal alcohol exposure (PAE) offspring. Representative immunoblots are shown below each figure. Subcellular fractions and anti-MR immunoreactivities were prepared as described in the Materials and Methods section. Data are presented as mean immunoreactivity corrected to Coomassie stain ± SEM in the PAE (n = 8–10) and SAC (n = 9–10) mice. Cytosolic MR levels were increased (p<0.05) in the PAE mice, whereas nuclear MR levels were not different in the two groups.

Both the GR and the MR have been shown to play roles in reversal learning [21]. We have shown that PAE is associated with reduced nuclear levels of the MR in the adolescent male mouse hippocampal formation [14] and, thus, we sought to determine if PAE altered nuclear MR levels in the mPFC. PAE and SAC nuclear MR levels were not different from each other (Figure 4B). This result demonstrates that the change in nuclear GR was not associated with a reciprocal change in MR levels in the PAE mouse mPFC, as was observed in the hippocampal formation [14]. However, cytosolic MR were elevated in the PAE mPFC (Figure 4A: t(12) = 2.32, p<.05). In the nuclear fraction, and to a lesser degree in the cytosolic fraction, two anti-MR immunoreactive bands were observed. These may represent different phosphorylated forms of the MR [22], [23].

### Cytosolic levels of FKPB 51, but not FKBP52, are elevated in the PAE mPFC

**Figure 5:**
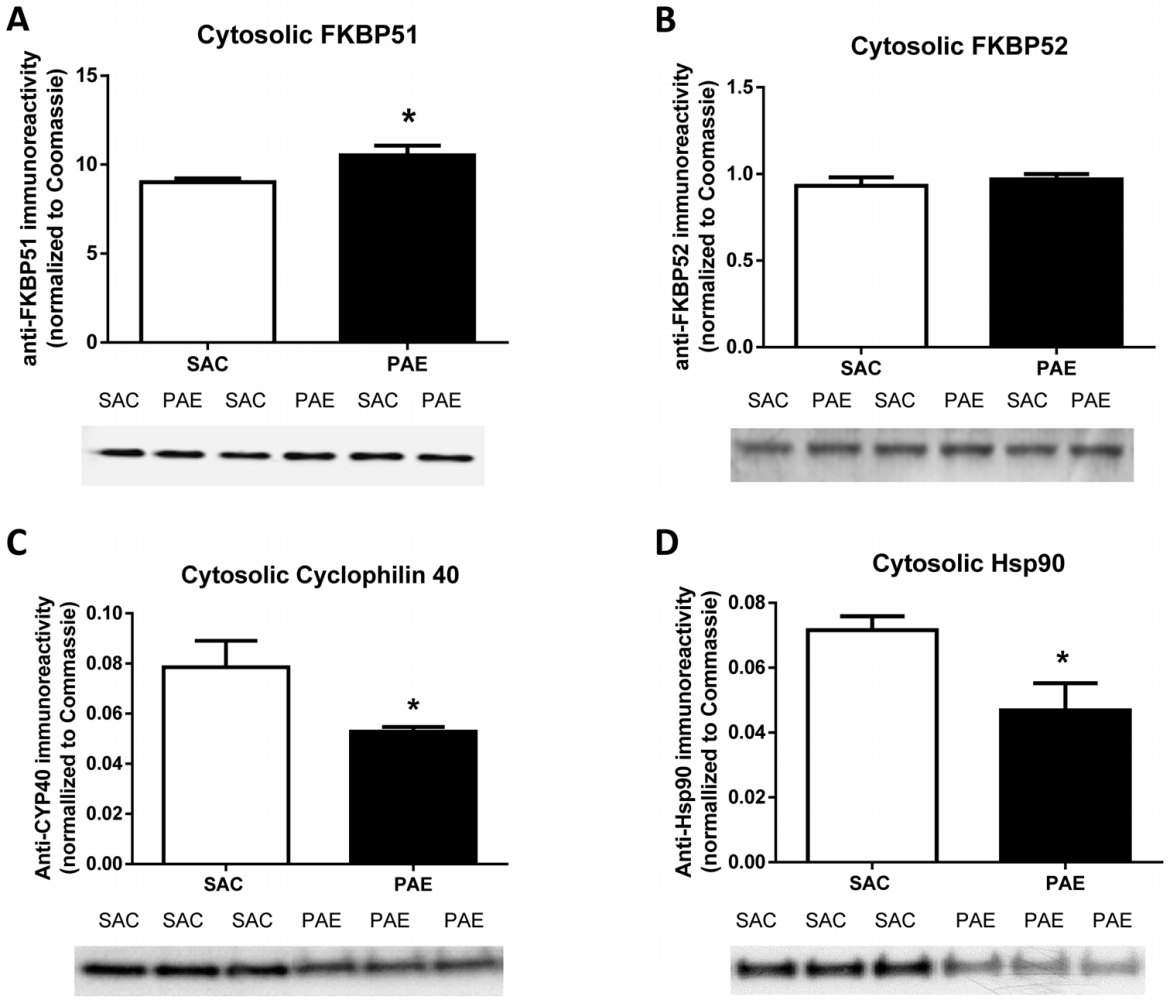
Cytosolic levels of the FKBP51 (A), FKPB52 (B), Cyclophilin 40 (C) and Hsp90 (D) in the medial frontal cortex of saccharin (SAC) control and prenatal alcohol exposure (PAE) offspring. Corresponding representative immunoblots are shown below each figure. Cytosolic fractions were prepared, and specific immunoreactivities were determined as described in the Materials and Methods. Data are presented as mean immunoreactivity corrected to Coomassie stain ± SEM in the PAE (n = 7) and SAC (n = 7) mice. Cytosolic FKBP51 (A) levels were increased (p = .02) and cytosolic FKBP52 (B) levels were not different between the PAE and SAC mice. Cytosolic cyclophilin 40 (C) levels were lower (p = .03) in the PAE offspring compared to SAC. Cytosolic Hsp90 (D) levels (p = .02) were lower in SAC mice.

As the binding of GR to FKBP51 favors a cytoplasmic localization of the complex, whereas its binding to FKBP52 enables nuclear trafficking [24], we reasoned that the reduction in mPFC nuclear GR could be associated with an increased level of cytosolic FKBP51 or, alternatively, a decreased level of cytosolic FKBP52. In order to test these possibilities, we measured the levels of FKBP51 and FKBP52 in a cytosolic fraction isolated from SAC and PAE mouse mPFC. Compared to SAC control samples, anti-FKBP51 immunoreactivity was significantly increased in PAE fractions (Figure 5A, t(12) = 2.59, p<0.02), whereas anti-FKBP52 immunoreactivities did not differ in SAC and PAE fractions (Figure 5B, t(12) = 0.90, ns). These findings are consistent with the observed decrease in nuclear localization of the GR.

### Cytosolic levels of Cyclophilin 40 (Peptidyl-prolyl cis-trans isomerase D) are reduced in PAE cytosolic fraction

Because of the observed differential effects of PAE on FKBP51 and FKBP52, we sought to determine whether PAE's effect on FKBP51 was unique or if another immunophilin was also affected. Thus, we assessed the effect of PAE on Cyclophilin 40 (CYP40), an immunophilin that binds Hsp90 via its tetratricopeptide repeat (TPR) domain and, like FKBP52, is involved in the transportation of GR to the nucleus [25]. FKPB51, FKBP52 and CYP40 compete for the same binding site on Hsp90, with FKBP51 displaying the highest affinity and CYP40 displaying the lowest affinity for Hsp90 [25]. Only a small percentage of the total cellular pool of Hsp90-GR heterocomplexes are bound to CYP40 [25]. Levels of CYP40 were lower in PAE cytosolic fractions compared to SAC (Figure 5C, [t (12)  = 2.4, p  = .034). Thus, although it is likely that CYP40 contributes a minor role to the localization of the total GR pool, the lower levels are consistent with reduced nuclear GR in the PAE mice.

### Cytosolic levels were lower but nuclear Hsp90 levels were not altered by PAE

Hsp90 promotes targeting of the GR to the nucleus via two mechanisms: it stabilizes the receptor in a conformation that favors high affinity ligand binding [26] and it binds FKBP52, which allows for association of the GR with the microtubule transport system [27]. Recent evidence also indicates that the Hsp90 chaperone system facilitates passage into the nuclear pore [27]. Further, an increase in trafficking of Hsp90 into the nucleus has been shown to decrease GR binding activity to the glucocorticoid response element (GRE) and has been linked to glucocorticoid resistance [28]. Thus, we assessed the effects of PAE on Hsp90 levels in the cytosol (Figure 5D) and the nucleus (Figure S3 in File S1). Cytosolic Hsp90 was significantly lower in the PAE compared to SAC [t(12)  = 2.63, p = .022] but levels in the nuclear fraction were not different [t (12)  = 0.34, ns]. The lower levels of Hsp90 in cytosol are consistent with reduced trafficking of GR to the nucleus and, thus, are consistent with the observed reduction in nuclear GR levels in PAE mPFC.

### Membrane levels of Dynamitin were lower in the PAE mice

**Figure 6:**
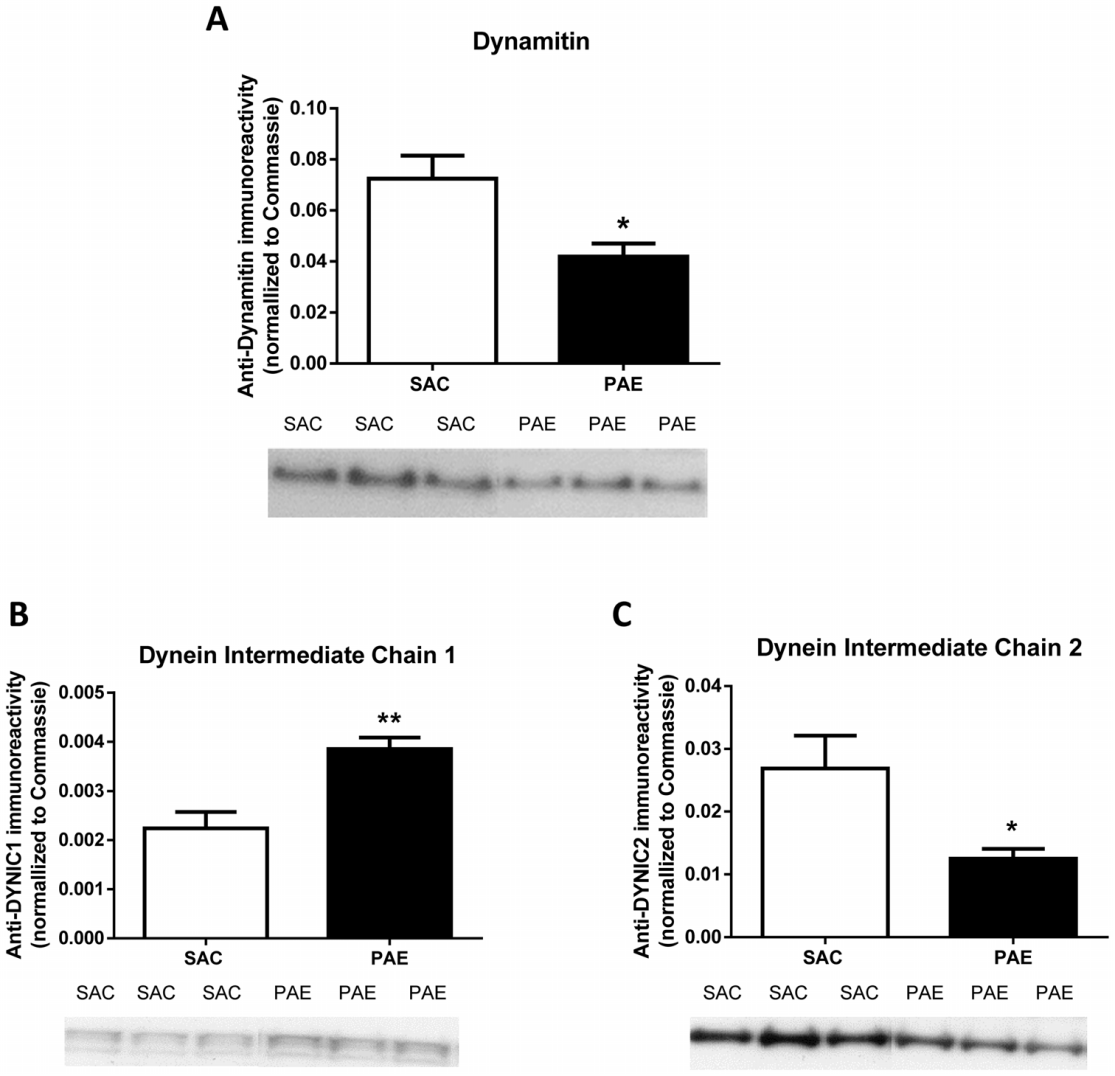
Membrane levels of dynamitin (A), Dynein IC1 (B) and Dynein IC2 (C) in the medial frontal cortex of saccharin (SAC) control and prenatal alcohol exposure (PAE) offspring. Corresponding representative immunoblots are shown below the respective figures. The cytosolic fraction was prepared and immunoreactivities were determined as described in the Materials and Methods. Data are presented as mean (± SEM) immunoreactivity corrected to Coomassie stain in PAE (n = 7) and SAC (n = 7) mice. Membrane dynamitin levels were lower (p = .01) in the PAE compared to SAC offspring. Membrane dynein C-1 levels were elevated (p = .002), while dynein IC-2 levels were lower (p = .012), in the PAE compared to SAC.

Transport of the GR from the cytosol to the nucleus is mediated by the microtubular system via FKBP52 binding of dynamitin [29], the central scaffolding subunit of the microtubule-associated dynactin complex [30]. This complex associates with the motor protein dynein and through this association is capable of transporting cargo to the nucleus. Because of its association with microtubules and other membrane components, dynamitin was assessed in the membrane fraction (Figure 6A). Levels of dynamitin were significantly lower in PAE compared to SAC membrane fraction [t (12)  = 2.96, p = .012], which is consistent with the observed reduction in nuclear GR levels in the PAE mice.

### Dynein IC1 was increased while Dynein IC2 level was decreased in the PAE mice

Dynein is a homodimer consisting of heavy, intermediate, light-intermediate, and light chains. It is the light-intermediate chains (IC) of dynein which are associated with the transport of GR into the nucleus. There are two light isoforms, IC1 and IC2, which have distinct membrane trafficking functions. Dynein IC2 regulates the nuclear translocation of GR [31], while dynein IC1 has been shown to be involved in the transport of other organelles and proteins [32]. We assessed the levels of dynein IC1 and IC2 in a post-nuclear lysate fraction in the PAE and SAC mice (Figure 6 B and C) and found a significant increase in levels of dynein IC1 (Figure 6B, t (12)  = 3.97, p = .002) and a significant decrease in dynein IC2 (Figure 6C, t(12)  = 2.63, p = .022) in the PAE. Lower levels of dynein IC2 are consistent with reduced translocation of GR into the nucleus.

## Discussion

In these studies we have found significant deficits in Y-maze reversal learning, a frontal cortical-dependent behavior, in PAE mice. Additionally, PAE was associated with reduced nuclear levels of the GR and reductions in GR trafficking proteins within the mPFC. The reduction in GR nuclear localization may be linked to the inability of PAE mice to demonstrate a flexible response to changes in stimuli saliency and contingency, as the GR has been shown to play a role in reversal learning [21].

An important role of the mPFC is to inhibit unnecessary associations and shift the attention towards stimuli which are predictive [2]. The reversal learning task is a measure of executive control, as it requires attention, working memory, and response inhibition. Failures in reversal learning indicate inflexible cognitive responding [33]; thus, the delay in reversal learning in the PAE mice indicates an inflexible response.

The GR shuttles between the cytosolic and nuclear compartments in a heterocomplex that is comprised of chaperone and co-chaperone proteins [34]. Movement between these compartments is controlled by the composition of the complex and its association with cytoskeletal motor proteins [35]. We found that nuclear GR levels were reduced in the mPFC of PAE mice. Collectively, our findings of increased cytosolic FKBP51 and reduced cytosolic Hsp90 and cyclophilin 40, combined with reductions in dynamitin and dynein IC2, support a model in which the reductions in nuclear GR levels are the result of decreased trafficking of the GR from the cytosol to the nucleus. However, we cannot exclude effects of PAE on mechanisms involved in nuclear import [7], nuclear retention [36] and nuclear export [37] of the GR. Additionally, intracellular corticosterone levels are an important driver of nuclear GR shuttling; analysis of the effects of PAE on tissue steroid hormone levels is an avenue for further research in our laboratory.

As the majority of cytosolic GRs are present in heterocomplexes that contain Hsp90 [38], the observed reduction in cytosolic Hsp90 levels in PAE mice is predicted to lead to reduced formation of mature, Hsp90-containing, GR complexes in the cytosol. It is these complexes which, upon ligand binding, associate with FKBP52 and translocate to the nucleus via the microtubule system [35]. Thus, the reduction in cytosolic Hsp90 is likely to contribute to the observed reduction in nuclear GR in the PAE mouse mPFC.

The presence of increased FKBP51 in PAE mouse cytosol is also predicted to contribute to the observed reduction in nuclear levels of GR, as the result of promoting the formation of cytosolic FKBP51 – GR complexes at the expense of FKBP52-containing complexes. This prediction is based on the proposal that the FKBP51:FKBP52 ratio is a primary regulator of the composition of GR complexes and subcellular localization. Barent and colleagues [39] reported that, in the presence of FKBP51, FKBP52 and CYP40, GR-Hsp90 complexes bind preferentially to FKBP51. Banerjee and colleagues [40] reported that FKBP51 over-expression in WCL2 cells, a cell line derived from CHO cells, produced increased cytosolic localization of the GR. Although several studies support the FKBP51:FKBP52 ratio model for the control of GR complex formation, subcellular localization and action, it is likely that there also are cell/tissue-specific mechanisms that contribute to the control of GR signaling [40]. It is interesting to note that alterations in FKBP51 levels have been implicated in psychiatric disorders, including depression [41], which have a higher incidence in FASD populations [42].

Translocation of the GR from the cytosol to the nucleus is mediated via the microtubule system. For the PAE mice, the reduction in dynein IC2 (Figure 6C) may represent a critical deficit leading to reduced levels of nuclear GR, as dynein IC2 is tasked with GR retrograde translocation [31]. The lower levels of dynamitin in the PAE mice combined with reduced dynein IC2 strongly point to a cause for the lower levels of nuclear GR.

The data present an interesting subcellular regulation mediated by PAE. Within the cytoplasmic faction we found an increase in cyp40 and FKBP51 but a decrease in the motor proteins (dynamitin and dynein IC2) involved in the nuclear trafficking of the GR co-complex. While the overall effect of these changes leads to decrease nuclear localization, the mechanisms through which PAE is able to differentially alter the expression of these proteins is currently unknown, but epigenetic regulation of gene expression is a possibility [43], [44]. Zhou et al observed a number of genes differentially regulated by early prenatal alcohol exposure, suggesting a complex interaction.

The role of the phosphorylation state of nuclear GR is still debated, but evidence suggests that the serine phosphorylation sites of GR that were analyzed in this study play a role in transcriptional targeting [19]. The absolute levels of each isoform (pS203 GR, pS211 GR and pS226 GR) were not different in PAE and SAC mice, suggesting that the basal transcriptional activity of the GR directed by these phosphorylation sites is likely not altered by PAE. However, the reduced level of total GR in the nucleus suggests that the pool of GR isoforms available for intra-nuclear conversion to one of the phosphorylated isoforms that we measured [45] is decreased and, thus, the ability to respond rapidly to a stimulus may be compromised. One possible explanation for the maintenance of the same absolute level of phospho GR species, while total GR levels are decreased, is that the levels of the kinases and phosphatases that control basal phosphorylation states of these residues are limiting and are fully active even in the presence of reduced total GR levels. The sites which we assessed are phosphorylated by several kinases [46] and dephosphorylated by several phosphatases [47], [48]. Alternatively, the relative increase in GR phosphorylation could be related to the reduced level of Hsp90, as reduced interaction between Hsp90 and GR is associated with increased levels of pS203 and pS226 GR, likely as the result of reduced association of the phosphoprotein phosphatase 5 (PP5) with the GR [47]. We think that this is an interesting area of research and we have initiated studies to begin addressing this issue.

We did not find differences in nuclear MR levels in SAC and PAE mice. Although the MR has been implicated in processes underlying reversal learning [21], it seems unlikely that the observed deficit in reversal learning in PAE mice is directly mediated via changes in MR-dependent gene expression. However, it is possible that the reduced nuclear GR:MR ratio, resulting from the decrease in GR levels with no change in MR levels, in PAE mice may affect reversal learning [21]. It is also possible that the MR plays an indirect role in the observed reversal learning deficit, as the observed increase in cytosolic MR may effectively reduce nuclear localization of the GR due to competition for binding to FKBP52-Hsp90 heterocomplexes.

The dorsomedial and prelimbic areas of the mPFC provide inhibitory inputs, while the ventromedial prefrontal cortex provides stimulatory input, to the HPA. These corticolimbic inputs are integrated within the bed nucleus of stria terminalis, which then sends afferents to the paraventricular nucleus of the hypothalamus to regulate the HPA [49]. In our PAE mouse model we have reported that hypothalamic levels of corticotropin releasing hormone (CRH) are increased [14]. PAE has also been associated with an increase in hypothalamic CRH by other investigators [50]. The decrease in the level of total nuclear GR in the mPFC of PAE mice may lead to less inhibitory control over the hypothalamic PVN by the mPFC and, thereby, contribute to the observed increase in CRH.

There are several studies demonstrating that postnatal maternal care can significantly alter the HPA axis and expression of both hippocampal and frontal cortical GR [51]. It is possible that the prenatal ethanol exposure, while it is withdrawn from the dam very shortly following birth, could affect maternal care and thereby GR in the offspring. In our development of this alcohol exposure paradigm, maternal care measures (pup retrieval time, time on nest, etc.) were assessed and not found to be different [15]. While these findings suggest no gross differences in maternal care, more detailed assessments might reveal subtle differences between the treatment conditions.

In conclusion, the data present a picture of the PAE mPFC having reduced nuclear GR capacity and potentially reduced trafficking of the GR to the nucleus. Thus, PAE animals may not be able to accommodate the demands placed on the GR system by challenging conditions, such as a change in stimulus saliency or contingency in a behavioral paradigm (e.g., reversal learning). This lack of capacity within the GR signaling system leads to perseveration in the reversal task. The link between GR trafficking and reversal learning is correlational at this point and confirmation of a direct association will require further study. The findings in this study, combined with our previous studies [14], indicate that the regulation of the subcellular localization of the GR is a common target for PAE in the brain. The effects of PAE on the GR system are brain region specific, as they differ between the mPFC and hippocampal formation, although alterations in subcellular trafficking may represent a common, underlying mechanism and, thus, represent an important therapeutic target. The mechanisms underlying the effects of PAE on trafficking proteins are a direction for future research.

## Supporting Information

File S1 This file contains: Figures S1–S3.
(DOCX)
