# Supplementary material for: Prenatal Alcohol Exposure Modifies Glucocorticoid Receptor Subcellular Distribution in the Medial Prefrontal Cortex and Impairs Frontal Cortex-Dependent Learning
Source: PLoS One. 2014 Apr 22;9(4):e96200. doi: 10.1371/journal.pone.0096200 (PMC3995983; doi:10.1371/journal.pone.0096200)
Supplement: File S1 — This file contains: Figures S1–S3. (DOCX) [file pone.0096200.s001.docx]

**Supplemental Figures**

Prenatal alcohol exposure alters glucocorticoid receptor trafficking in the medial frontal cortex of adolescent mice.

Andrea M. Allan, Samantha L. Goggin, Kevin K. Caldwell

Department of Neurosciences, School of Medicine, University of New Mexico Health Sciences Center, Albuquerque, New Mexico, 87131, USA

Supplemental figure 1

Supplemental Figure 1: Demonstration of the linearity of the Coomassie stain. Range tested is from 5-60µg of protein. Study used less than 35 µg of protein throughout.

Supplemental figure 2

B

A

C

D

Supplemental Figure 2- Demonstration that Commassie staining is not altered in the PAE relative to SAC in the membrane fraction (A) post nuclear membrane (B) cytosolic (C) or nuclear fractions (D). Preparation of these fractions are described in the Materials and Methods section Data are mean (±SEM) optical density units, n=7 per treatment condition.


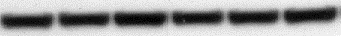


Supplemental Figure 3: Nuclear levels of Hsp90 in the medial frontal cortex of saccharin (SAC) control and prenatal alcohol exposure (PAE) offspring. Nuclear fractions were prepared, and anti-Hsp90 immunoreactivities were determined, as described in the Materials and Methods. Data are presented as mean antiHSP90 immunoreactivity corrected to Coomassie stain ± SEM. Nuclear Hsp90 levels (B) were not different between the PAE and SAC mice. The first 3 lanes are SAC and the last 3 lanes are PAE.
